# PD69 Mental Health And The Use Of Antidepressants In Brazilian Media: A Content Analysis Using Natural Language Processing Tools

**DOI:** 10.1017/S0266462325102018

**Published:** 2025-12-29

**Authors:** Sabrina Wolenski Bartoszewski, William Jorge Pereira, Aline Verderamis, Cristiane De Cassia Bergamaschi Motta, Silvio Barberato-Filho, Luciane Lopes, Luis Gustavo Modelli de Andrade, Juliana Machado-Rugolo

## Abstract

**Introduction:**

Despite the significant impact of the media on individuals with mental illness, newspaper articles related to antidepressants have not been systematically studied. The present study aimed to analyze Brazilian journalistic coverage on the use of antidepressants to understand how the news presents and shapes the topic of antidepressants for its readers.

**Methods:**

This qualitative study evaluated journalistic content on antidepressant use from Folha de São Paulo, a newspaper available in digital format. Articles published between 1 January 2019 and 31 December 2023 were collected and stored in a database for analysis. Natural language processing (NLP) techniques, combined with machine learning, were applied. The R software (version 4.3.3) with tidytext, tm, tidyverse, and stringr packages was used for the analyses. The study identified patterns and trends in language usage, focusing on frequently occurring terms to understand how antidepressants are portrayed in media content.

**Results:**

The initial research was conducted using the keyword “antidepressants” in the search engine of the Folha de São Paulo. Of the articles assessed for eligibility, 182 were included in the study. Across all the articles, the analysis aimed to identify the most frequently used words, resulting in a word cloud. The most used words in the text body were “treatment,” followed by “years,” “anxiety,” “women,” and “pandemic.” Regarding the most frequent words in the titles, they were “depression,” “health,” and “study.” On average, there was no significant change in the total number of n-grams used.

**Conclusions:**

The findings illustrated how Brazilian media frames antidepressant use, revealing potential misinformation or stigma. Understanding these representations can guide strategies for improving public awareness and reducing stigma around mental health. By highlighting trends in journalistic narratives, this study contributes to public health policies that promote accurate, responsible communication about antidepressant use.

